# Supplementary material for: A yeast phenomic model for the influence of Warburg metabolism on genetic buffering of doxorubicin
Source: Cancer Metab. 2019 Oct 23;7:9. doi: 10.1186/s40170-019-0201-3 (PMC6806529; doi:10.1186/s40170-019-0201-3)
Supplement: Supplementary file 9 — Additional file 9. GO term-specific heatmaps for REMc/GTF-enriched clusters. GO term-specific heatmaps for significant GO process terms were generated as described in methods and Figs. 3 and 4. Any related child terms are presented in subsequent pages of the parent file name. GO terms with more than 100 children, with 2 or fewer genes annotated to the term, or a file size over 300KB are not shown. All heatmaps are generated with the same layout (see Figs. 3 and 4). [file 40170_2019_201_MOESM9_ESM.bz2 › Additional_File9_GOTermHeatmaps/Additional_File9_GOTermHeatmap/translation.pdf]

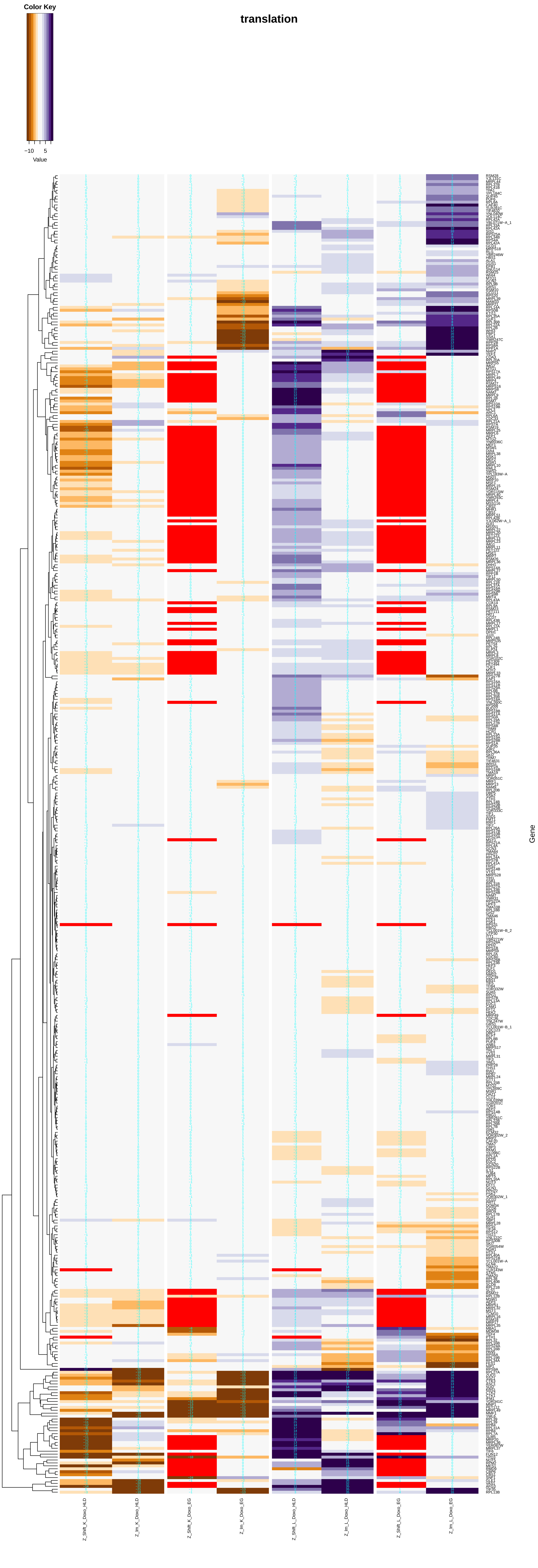
translation
Color Key
−10
5
Value
Z_Shift_K_Doxo_HLD
Z_lm_K_Doxo_HLD
Z_Shift_K_Doxo_EG
Z_lm_K_Doxo_EG
Z_Shift_L_Doxo_HLD
Z_lm_L_Doxo_HLD
Z_Shift_L_Doxo_EG
Z_lm_L_Doxo_EG
Gene

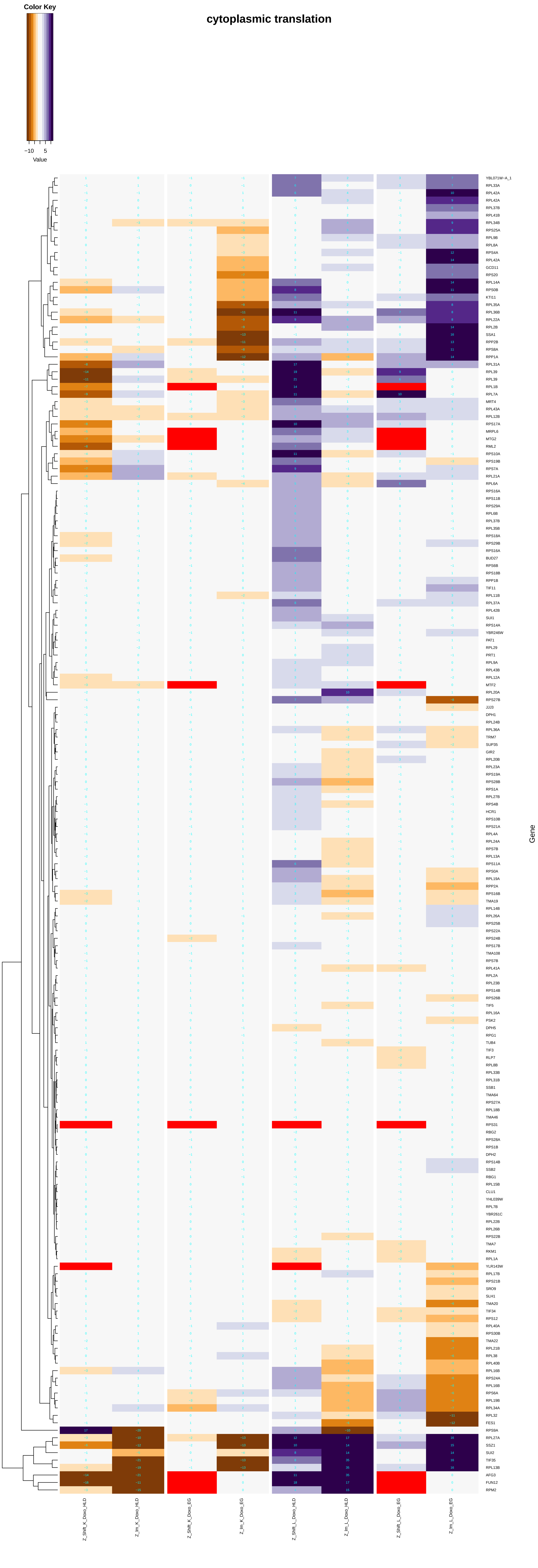
cytoplasmic translation
Color Key
−10
5
Value
Gene
Z_Shift_K_Doxo_HLD
Z_Im_K_Doxo_HLD
Z_Shift_K_Doxo_EG
Z_Im_K_Doxo_EG
Z_Shift_L_Doxo_HLD
Z_Im_L_Doxo_HLD
Z_Shift_L_Doxo_EG
Z_Im_L_Doxo_EG

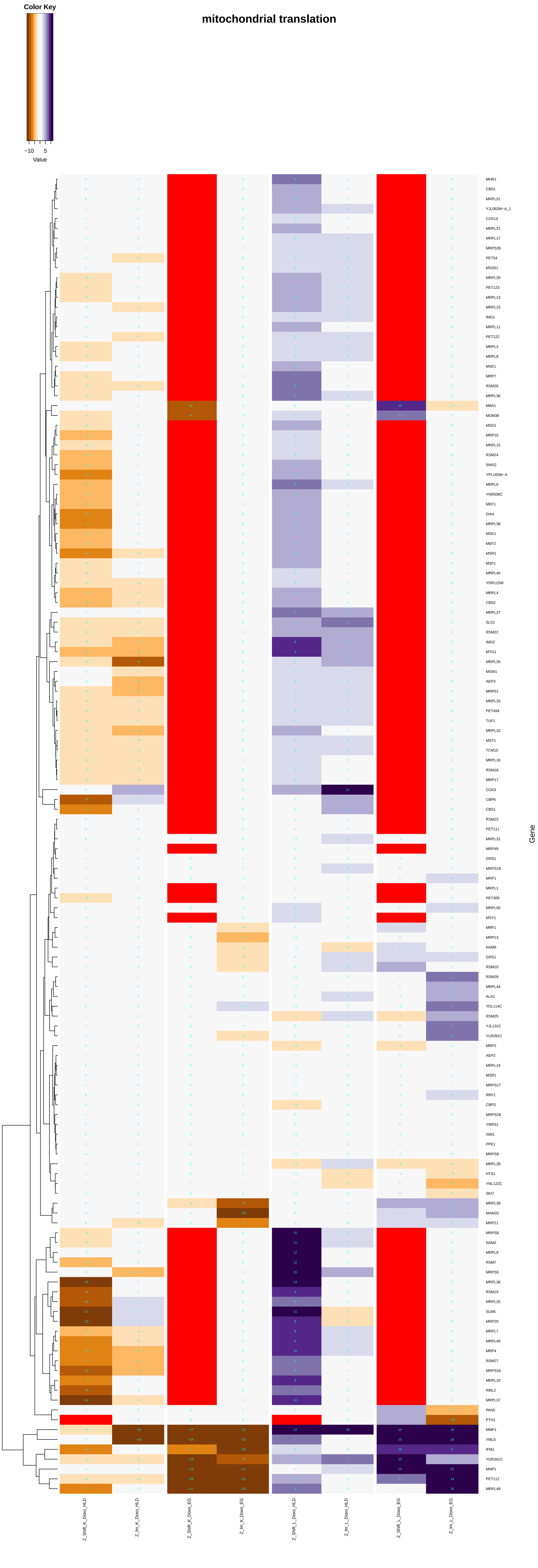
mitochondrial translation
Color Key
−10 5
Value
MHR1
CBS1
MRPL51
YJL062W−A_1
COX14
MRPL22
MRPL17
MRPS35
PET54
MSS51
MRPL20
PET123
MRPL13
MRPL23
IMG1
MRPL11
PET122
MRPL3
MRPL8
MSE1
MRP7
RSM26
MRPL36
MBA1
MDM38
MSD1
MRP10
MRPL15
RSM24
SWS2
YPL183W−A
MRPL6
YNR036C
MEF1
DIA4
MRPL38
MSK1
MEF2
MSR1
MSF1
MRPL40
YDR115W
MRPL4
CBS2
MRPL27
SLS1
RSM22
IMG2
MTG1
MRPL35
MSW1
AEP3
MRP51
MRPL33
PET494
TUF1
MRPL32
MST1
TCM10
MRPL16
RSM18
MRP17
COA3
CBP6
CBS1
RSM23
PET111
MRPL31
MRP49
GRS1
MRPS18
MRP1
MRPL1
PET309
MRPL50
MSY1
MRF1
MRP13
NAM9
GRS1
RSM10
RSM28
MRPL44
ALA1
YOL114C
RSM25
YJL131C
YLR281C
MRP2
AEP2
MRPL24
MSR1
MRPS17
RRF1
CBP3
MRPS28
YMR31
ISM1
PPE1
MRPS9
MRPL28
HTS1
YNL122C
SKI7
MRPL39
MAM33
MRP21
MRPS8
NAM2
MRPL9
RSM7
MRPS5
MRPL36
RSM19
MRPL25
SLM5
MRP20
MRPL7
MRPL49
MRP4
RSM27
MRPS16
MRPL10
RML2
MRPL37
PAN5
PTH1
MMF1
YML6
IFM1
YDR341C
MNP1
PET112
MRPL49
Gene
Z_Shift_K_Doxo_HLD
Z_Im_K_Doxo_HLD
Z_Shift_K_Doxo_EG
Z_Im_K_Doxo_EG
Z_Shift_L_Doxo_HLD
Z_Im_L_Doxo_HLD
Z_Shift_L_Doxo_EG
Z_Im_L_Doxo_EG
